# Impact of glycan cloud on the B-cell epitope prediction of SARS-CoV-2 Spike protein

**DOI:** 10.1038/s41541-020-00237-9

**Published:** 2020-09-04

**Authors:** René Wintjens, Amanda Makha Bifani, Pablo Bifani

**Affiliations:** 1 Unit of Microbiology, Bioorganic and Macromolecular Chemistry, Department of Research in Drug Development (RD3), Faculté de Pharmacie, Université Libre de Bruxelles, 1050 Brussels, Belgium; 2 Programme in Emerging Infectious Diseases, Duke-NUS Medical School, Singapore, 169857 Singapore; 3 Singapore Immunology Network (SIgN), A*STAR, 8A Biomedical Grove, Immunos Building, Singapore, 138648 Singapore; 4 Infectious Diseases Programme and Department of Microbiology and Immunology, Yong Loo Lin School of Medicine, National University of Singapore, Singapore, 119077 Singapore; 5 Department of Infection Biology, Faculty of Infectious and Tropical Diseases, London School of Hygiene and Tropical Medicine, London, UK

**Keywords:** Vaccines, Vaccines

## Abstract

The SARS-CoV-2 outbreak originated in China in late 2019 and has since spread to pandemic proportions. Diagnostics, therapeutics and vaccines are urgently needed. We model the trimeric Spike protein, including flexible loops and all N-glycosylation sites, in order to elucidate accessible epitopes for antibody-based diagnostics, therapeutics and vaccine development. Based on published experimental data, six homogeneous glycosylation patterns and two heterogeneous ones were used for the analysis. The glycan chains alter the accessible surface areas on the S-protein, impeding antibody-antigen recognition. In presence of glycan, epitopes on the S1 subunit, that notably contains the receptor binding domain, remain mostly accessible to antibodies while those present on the S2 subunit are predominantly inaccessible. We identify 28 B-cell epitopes in the Spike structure and group them as non-affected by the glycan cloud versus those which are strongly masked by the glycan cloud, resulting in a list of favourable epitopes as targets for vaccine development, antibody-based therapy and diagnostics.

## INTRODUCTION

A cluster of viral pneumonia emerged in Wuhan, Hubei Province, China in December 2019. Shortly after, the aetiological agent was determined to be a novel coronavirus now referred to as Severe Acute Respiratory Syndrome 2 (SARS-CoV-2) which causes Corona Virus Disease 2019 (COVID- 19). Since its emergence, SARS-CoV-2 has spread across the globe, establishing local outbreaks in over 200 countries^1^. Close to 17.5 million reported infections and over 675,000 death have been reported as of August 1^st^, and these numbers are likely greater due to insufficient testing in parts of the world and asymptomatic carriers^2^.

SARS-CoV-2 is a member of the betacoronavirus genus with a single stranded RNA genome. Encoded in the genome is the immunodominant Spike protein (S). The S-protein is translated as a single protein and subsequently cleaved into two subunits. Three S-proteins assemble on the surface of the virion to form a trimeric spike. The S1 subunit is involved in receptor recognition and binding, while the S2 subunit contains a fusion peptide and necessary machinery for fusion, typically observed in class I fusion proteins. The S-protein binds host receptor angiotensin converting enzyme 2 (ACE2) and mediates viral entry into the host cell, similar to 2002 SARS-CoV^3^. The receptor binding domain (RBD) of SARS-CoV and SARS-CoV-2 shares 72% identity at the amino acid level^4^. Thus, it is no surprise that some antibodies are species specific^5^; while other cross-reactive antibodies between SARS-CoVs have also been reported^6^. However, SARS-CoV-2 has a flexible loop which projects into the hydrophobic pocket of ACE2, conferring a stronger interaction with the receptor than observed for SARS-CoV^5,7^. While this S-protein protrusion out of the viral envelop facilitates receptor recognition and binding, it also leaves the protein exposed to recognition by the host immune system.

Consequently, the S1-subunit epitopes of SARS-CoV and SARS-CoV-2 are target candidates for induction of a B-cell response^8,9^. A candidate monoclonal antibody isolated from SARS-CoV patients during the 2002 outbreak can cross-neutralise SARS-CoV2^10^. Accordingly, potential SARS-CoV-2 epitopes have been suggested based on homologous regions of the SARS-CoVs S-protein that are known to be immunogenic^8,9^. Strictly relying on known SARS-CoVs epitopes shared with SARS-CoV-2 limits the selection of antigenic sequences to non-SARS-CoV-2 specific epitopes^8,9^. A limitation of these published studies is the failure to consider the impact of glycosylation, which could shield some of the selected epitopes.

As the number of COVID-19 cases continues to increase around the world, there is an urgent need for diagnostics, therapeutics and vaccines. This may be hampered by heavy glycosylation of the SARS-CoV-2 S-protein trimer, hindering exposure to the host adaptive immune system. With the critical need for a vaccine, it is crucial to identify epitopes that are conserved, accessible and unrestricted by the glycan chains to elicit a robust neutralising antibody response from the host. Thus, we aimed to identify epitopes, which are readily available to the host immune system. We identify various epitopes in the S-protein, considering the protein structure, the flexible loops and stearic hindrance resulting from protein glycosylation in the hope that this may advance vaccine development and antibody-based therapies and diagnostics.

## RESULTS

### N-glycosylation sites in the SARS-CoV-2 S-protein

**Fig. 1:**
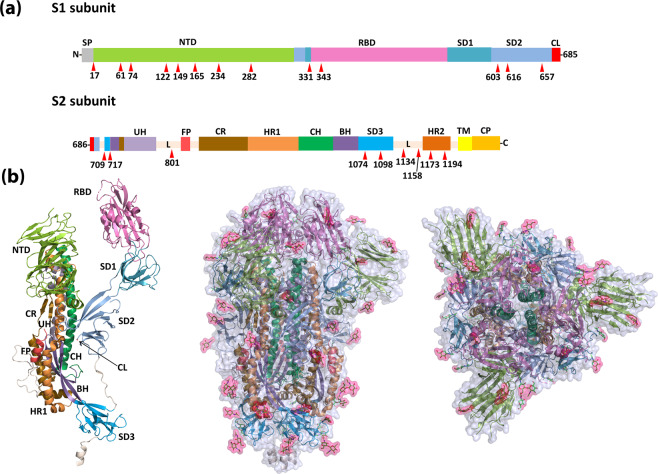
SARS-CoV-2 S-protein harbours numerous N-glycosylation sites. **a** Schematic diagram of the SARS-CoV-2 S-protein domain organisation. The structural domains are: SP signal peptide, NTD N-terminal domain, L linker region, RBD receptor-binding domain, SD subdomain, CL cleavage loop, UH upstream helix, FP fusion peptide, CR connecting region, HR heptad repeat, CH central helix, BH β-hairpin, TM transmembrane region, CP cytoplasmic part. The structural domains were defined as described Supplementary Fig. 1. N-glycosylated asparagine was labelled and localised in the schematic diagram by an arrow in red. **b** 3D representation of the prefusion SARS-CoV-2 trimeric S-protein solved by cryo-EM (PDB id 6VXX). Structural domains were coloured using the same colouring scheme of **a**, images for side and top view in left and right, respectively. Protein surface was depicted in grey and the N-acetylglucosamine moieties found in the cryo-EM structure was marked in pink.

Twenty-two experimentally determined N-glycosylation sites were evaluated to differentiate SARS-CoV-2 S-protein antigenic regions exposed to B cells from epitopes that are shielded by a glycan chain^11–13^. The 22 N-glycosylation positions are unevenly distributed across the two S-protein subunits, accounting for 66 sites in the trimeric structure (Fig. 1a and Supplementary Fig. 1). A 3D rendition of SARS-CoV-2 was generated with the decorating glycosylation sites as observed in the cryo-EM structure (PDB id 6VXX) (Fig. 1b). The distal domains, including the N-terminal domain (NTD) and the RBD are predominantly composed of β-sheets. As the protein approaches the envelope, the domains primarily adopt α-helix structures and account for most of the glycosylation sites. The 22-glycosylation sites and glycosyl groups considered here have been validated experimentally by cryo-EM and high-resolution mass spectrometry (LC-MS/MS) analysis^11–14^. Mass spectrometry analysis of recombinant S proteins produced in mammalian human embryonic kidney 293T (HEK293T) cells or insect cells confirmed the complete occupancy of the 22 sites^12,13^. A large percentage of these glycosylated residues (18/22) are conserved in SARS-CoV (Supplementary Fig. 2).

### Modelling the glycosylation pattern of the trimeric spike with the flexible loop regions

**Fig. 2:**
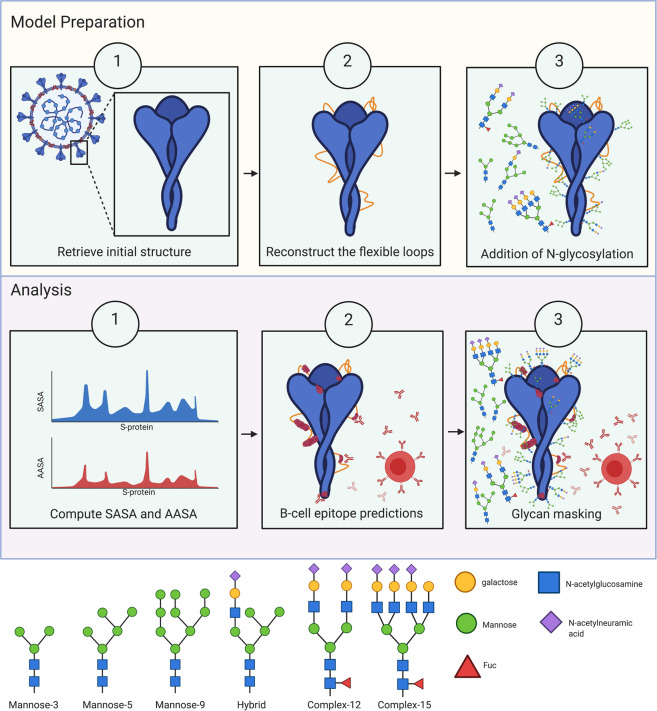
Antibody accessible surface area was determined by a structure-based approach that accounted for N-glycosylation patterns. **a** A schematic description of the model preparation steps taken to generate a 3D rendition of the SARS-CoV-2 trimeric spike with the N-glycosylation sites. **b** A schematic diagram of the approach taken to elucidate epitopes that remain accessible to antibody detection despite glycosylation of the S-protein. **c** A cartoon representation of the six glycan chains considered in the analysis of homogenously glycosylated models.

We sought to build a glycosylation pattern model of the SARS-CoV-2 S-protein to identify accessible antigenic epitopes that were not masked with glycosylation by (i) retrieving the trimeric S-protein from the protein data bank (PDB) (PDB id 6VXX; 2.80 Å resolution; segment 27-1147)^14^ (ii) reconstructing the flexible loops not present in the starting structure and (iii) model glycan chains attached on the N-linked glycosylation sites in order to be able to (iv) compute the solvent-accessible surface area (SASA) and antibody-accessible surface area (AASA) and (v) predict the B-cell epitopes and glycan masking (Fig. 2a). Six different N-glycan types were considered in our study to model homogenous glycosylation patterns, including three high-mannose N-glycan types (Man3, Man5, and Man9), one hybrid type (Hyb8). Two complex types (NAc(4)Man(3)Fuc(1)Gal(2)Neu(2) (Complex-12) and NAc(6)Man(3)Fuc(1)Gal(4)(Neu(3) (Complex-15)) were also included, the former of which was frequently identified in experimental data and the latter glycan served to illustrate the effects of a large glycan chains on epitope recognition restriction^11–13^ (Fig. 2b). Two additional heterogeneous glycosylation patterns were included, based on Watanabe et al.^12^ (pat1) and Shajahan et al. (pat2)^11^. However, it is important to note glycosylation patterns and glycan species can vary in different cell types^15^ and are altered in malignant cells compared to non-malignant cells^16^. As these previous S-protein glycosylation studies were performed in HEK293 cells^11,12^, which were immortalised by adenovirus, it is possible that the glycosylation pattern may not reflect a natural infected cell. Consequently, we chose to additionally include common glycosylation types found in proteins of healthy human cells. Noteworthy, these sugars are highly flexible dynamic structures which adopt various conformations and hence, rendering a single structural arrangement misleading when representing an *in vivo* structure. Here, we chose to use two different conformations to model each of the glycan chains.

### Profiling accessibility to the surface area

Eighteen glycosylation sites almost fully decorate the SARS-CoV-2 S-protein in glycan chains, creating a glycan cloud (Supplementary Fig. 3). The accessible surface area (ASA) was calculated in order to determine which protein regions remain accessible to antibody binding. Succinctly, FreeSASA^17^ was used to calculate the ASA for a de-glycosylated model, the cryo-EM structure, and the eight glycosylated models elucidated in this study (six homogenous N-glycan types and two composite patterns comprising each of ten models, i.e. five loop models times two glycan conformations). Firstly, the solvent-ASA (SASA) was calculated by using the probe radius of a water molecule to be used as reference. As expected, the SASA profile in the de-glycosylated and glycosylated models do not differ greatly (Supplementary Fig. 4). This is expected as water molecules can pass in between the glycan chains on the S-protein surface.

**Fig. 3:**
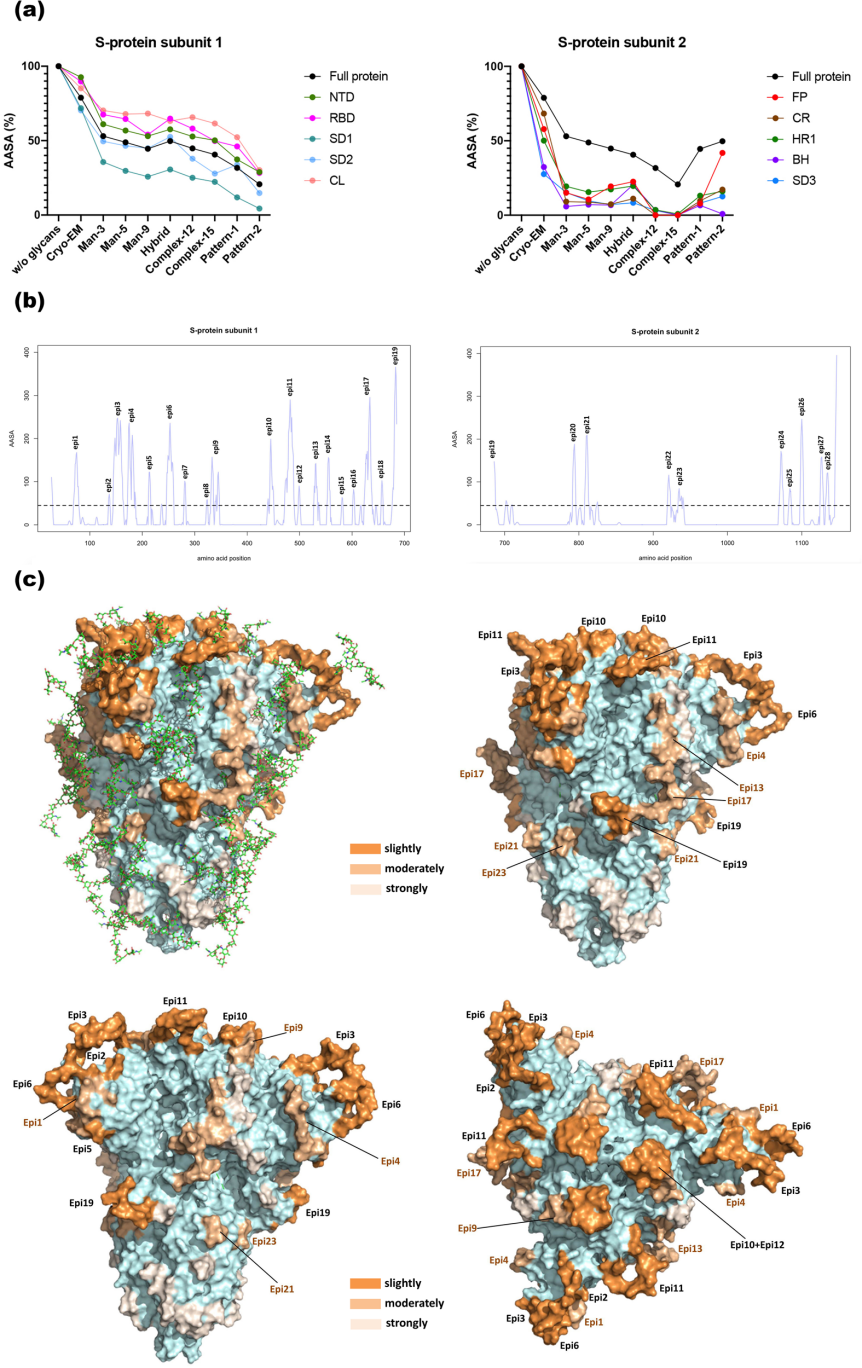
B-cell epitopes are differentially exposed under different glycan shielding effects. **a** Progression of AASA for each structure domain as a function of increasingly larger homogeneous glycan chains, 2 heterogeneous glycosylation patters, as well as two previously published glycosylation profiles^11,12^. AASA of each domain were expressed as a percentage in relation of the de-glycosylated model. The AASA of the total protein was also plotted for reference. **b** Profile of AASA defining the B-cell epitopes using a threshold of 45 Å (shown as a dotted horizontal line). Each peak has been labelled according to the corresponding epitope sequentially as in Fig. 4. **c** 3D representations of B-cell epitopes onto the protein surface. The epitopes were coloured according to the glycan shield effect (Fig. 4), epitopes slightly affected in dark orange (epi2, epi3, epi5, epi6, epi10, epi11, epi12 and epi19), epitopes moderately affected in light orange (epi1, epi4, epi9, epi9, epi17, epi21 and epi23), and the epitopes strongly affected in pale orange (epi7, epi8, epi14, epi15, epi16, epi18, epi20, epi22, epi24, epi25, epi26, epi27 and epi28). The epitopes slightly and moderately affected by glycan presence were labelled. The hybrid glycan chains were shown in stick representation in the upper-left image.

The antibody-ASA (AASA) was subsequently computed using a large probe radius comparable in size to the recognition domain of an antibody, allowing surface points available for protein-protein contacts, such as antibody-antigen^18^ to be identified. Unlike the SASA profiles, several domains of the S-protein were not accessible following glycosylation (Fig. 3a). Notably, the antibody accessibility of the S1 subunit (the NTD, subdomain 1 (SD1), subdomain 2 (SD2) and cleavage loop (CL)), were less affected by the glycosylation as was the case for the RBD and CL which were only partially obstructed by glycosylation (Fig. 3a). In contrast, except for the fusion peptide (FP), the AASA of all structural domains in the S2 subunit decreased rapidly in relation with the presence of glycans of increasing size, with the upstream helix (UH) and connecting region (CR) being the most affected domains and the central helix (CH) found to be completely inaccessible to antibodies even in the absence of glycans (Fig. 3a and Supplementary Table 1). The AASA of the total protein was estimated at 44% with a glycosylation pattern from Watanabe et al.^12^, of which three domains, namely NTD, RBD and CL retained about 30% of the antibody accessibility, even in the presence of the large complex chain Complex-15 (Fig. 3a). While the conserved AASA for RBD and CL could result from their function as receptor binding and cleavage site respectively; a plausible function for the protein-accessibility of NTD is less clear. Importantly, the AASA was not more impacted in the presence of longer glycan chains, likely due to the outwards orientation of the chains. These longer glycan chains project further outwards rather than folding back into the protein obstructing additional sites.

### B-cell epitope prediction and glycan shield impact

**Fig. 4:**
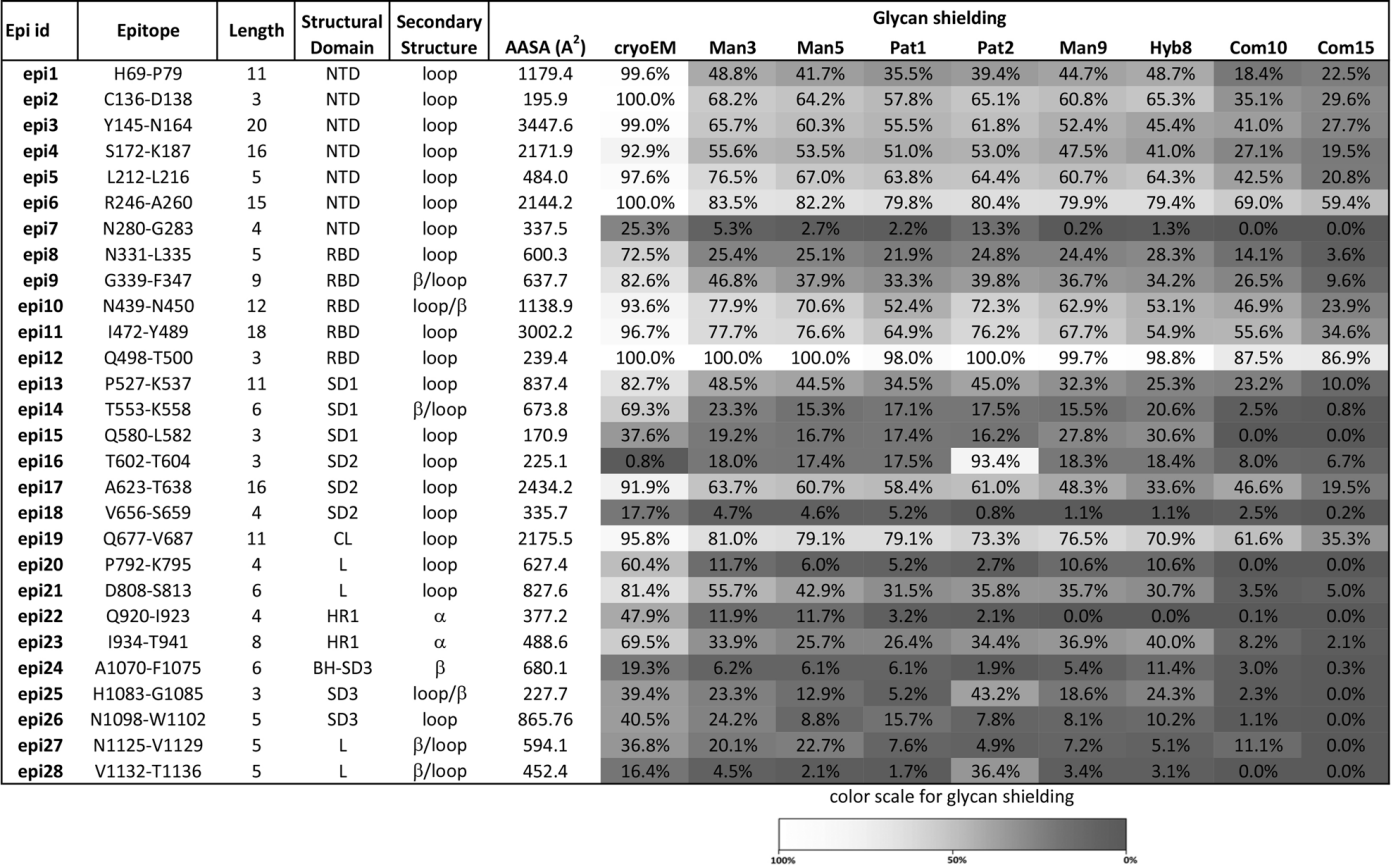
Several predicted B-cell epitopes are partially or fully shielded by glycan chains. Figure depicting the 28 B-cell epitopes predicted based on the S-protein trimeric spike structure. Epitope region, length, position in the spike protein and 3D structure are shown. The percentage of antibody-accessible surface area of said epitopes under different glycosylation patterns is also presented. “CryoEM” is for the glycosylation pattern found in the initial S trimer structure and the labelling of other glycan chains are defined in Methods. The glycan chains are arranged in ascending order of their global SASA in the models (see Supplementary Table 5).

Based on the AASA profile of the de-glycosylated model (Fig. 3b), we identified 28 protruding epitopes and evaluated their accessibility based on position and glycan shielding (Fig. 4). As expected most of epitopes were localised in the loop regions and 42% (12/28 epitopes) were found within NTD and RBD; the two critical domains for antibody recognition. In contrast, the epitopes in the S2 domain or the C-terminal close to the membrane were shielded by a glycan cloud. Accordingly, the AASA values of these latter epitopes were very low, dropping close to zero with the largest glycans (<10%) (Fig. 4). The 28 predicted B-cell epitopes were categorised into three groups based on the degree of accessibility lost upon glycosylation with sugar of increasing size: slightly shielded (epi2, epi3, epi5 and epi6, in the NTD; epi10, epi11 and epi12 in the RBD; and epi19 in the CL); moderately (epi1, epi4 in the NTD; epi9 in the RBD, epi13 in the SD1; epi17 in the SD2; epi21 and epi23) or strongly masked (epi7, epi8, epi14, epi15, epi16, epi18, epi20, epi22, epi24, epi25, epi26, epi27 and epi28) (Fig. 3c). All the slightly and moderately shielded epitopes were localised within the S1 domain or N-terminal part of the protein, except epi21 and epi23. This underlines the importance of S1 domain for antibody recognition. It is critical to highlight that the loop spanning positions 828-853, was not modelled in this study and may likely harbour additional epitopes. Furthermore, conformational epitope (discontinuous epitopes), that form when distal parts of the amino acid sequence assemble in the tertiary structure, are found here comprising of the following clusters, epi1-epi2-epi-5-epi6, epi3-epi6, epi4-epi5, and epi10- epi12, among the epitopes less affected by glycosylation (Fig. 3c and Supplementary Table 2).

## DISCUSSION

The COVID-19 pandemic has spread rapidly worldwide. It has exhausted health systems, burdened the global economy and brought many countries to a standstill. The international community is now looking to researchers to alleviate the impacts of SARS-CoV-2 through potential drugs, antibody therapy and most importantly, novel vaccine candidates. Here, we accounted for the N-glycosylation sites in different conformations on the SARS-CoV-2 trimeric S-protein in order to identify promising antibody accessible epitopes.

Recent published experimental data shows that the SARS-CoV-2 trimeric spike is highly glycosylated^12,13^. The degree of glycosylation is a key feature to consider when selecting potential B-cell epitopes and designing antibody-based diagnostics, therapeutics or vaccines, as the number of accessible epitopes is limited. Noteworthy, is the differential distribution of these glycan along the protein. The S2 subunit glycosylation is more concentrated, especially towards the membrane while the S1 subunit contains more exposed domains possibly due to accessibility required for RBD and CL (Fig. 1a).

We determined that the epitopes in the S2 subunit become completely shielded by the glycan cloud, whereas this phenomenon is not observed with epitopes localised to the S1 subunit. This may be explained by the fact that the S2 subunit is a more compact structure, consisting mostly of α-helices (Fig. 1b), thereby generating a denser glycan cloud (Fig. 3c). The biological reasons why the S2 subunit is more protected by glycans remains to be determined. In a marked contrast, the subunit S1 has a more extended structure, with several β-sheets, creating a diffuse glycan cloud mitigating shielding effect of the epitopes (Fig. 3c).

We modelled the N-glycosylation pattern garnishing the S-protein in its trimeric conformation considering the flexible loop regions which were absent in the published experimental cryo-EM structure^14^. Our modelling approach, however has a few limitations. First, as glycan chains are dynamic structures, a single confirmation is insufficient to represent the complex structure. Thus, our model benefits from using two glycan chain conformations to account for the dynamic nature of N-glycosylation. Despite this approach, one has to bear in mind the flexibility of the glycan chains, as well as of that of the loops. In order to account the variability associated with the flexibility of the loops, here five different loop conformations have been considered in the analysis. A more realistic model based on molecular dynamics simulations would necessitate huge computational resources. Second, we based our model strictly on the closed structure (triple down protomers of the RBD) of the S-protein, rather than the open structure observed during receptor binding^19^. Nevertheless, this model still allowed us to capture a previously identified epitope S230-CoV-1^5,19^ from the open conformation, that corresponds in part to the epi11, supporting the validity of our model.

Our model is in-part corroborated by recent reports of experimentally determined epitopes^10,20–22^ (Supplementary Table S5). For example, three neutralising monoclonal antibodies COV2-2130, COV2-2165 and COV2-2196 contain respectively the following key residues K444 and G447 (epi10), N487 and F486 (epi11), and N487 found in epi11 as well^23^. Likewise, 15/17 epitopes reported by Ravichandran and colleagues were identified in our work^21^. Another study using sera from COVID-19 convalescent patients led to the identification of two immunogenic peptides derived from the SARS-CoV-2 S-protein^24^. These two immunogenic peptides, S14P15 (region 553-570, SD1, partially impacted by glycan shield), and S21P2 (region 809-826, FP, moderately impacted by glycan shielding), correspond to epi14 and epi21, respectively, described in this study (Fig. 4). Other previously reported structures of the SARS-CoV S-protein RBD in complex with the three antibodies, namely 80R (PDB id 2GHW)^25^, m396 (PDB id 2DD8)^26^ and F26G19 (PDB id 3BGF)^27^, show the involvement of epitopes corresponding to SARS-CoV2 epi10 to epi12 antibody recognition sites found here. The partial match between our RBD epitopes and the antibody-binding regions in SARS-CoV RBD emphasises the dissimilarities of the two SARS-CoVs RBDs as confirmed by the poor cross-reactivity of these three antibodies^28^. Nevertheless, cross-reactivity has been reported with the monoclonal antibody S309 derived from memory B-cells from a 2003 SARS-CoV convalescent patient that recognises discontinuous eptiopes^10^. Our model correctly identified fragments 333-346 and 440-441 (corresponding to epi9 and epi10) which are approximately 25 Å apart, but did not identify fragment 354-361 in the discontinuous epitopes recognised by antigen S309^10^ (Supplementary Table 3). The majority of discontinuous epitopes are composed of 1-5 linear segments comprising of 1-6 amino acids. As such we have provided a distance matrix highlighting the space between the 28 epitopes identified here (Supplementary Table 2). The model presented here is in agreement with others linear-based and discontinuous epitope predictions^29,30^. Noteworthy, the interaction between RBD and ACE2 involves residues 445-456, 473-477, and 484-505 corresponding to our epi10, epi11 and epi11 + epi12, respectively^31^.

The glycan masking of epitopes may address why some patients’ sera is limited in its ability to prevent pseudovirus entry into host cells^14^. A similar epitope masking phenomenon has been observed in related alphacoronavirus, NL63 by cryo-EM^32^ as well as human immune deficiency virus (HIV), where the receptor binding proteins are masked by heavy glycosylation shielding antigen exposure referred to as a glycan shield^33^. De-glycosylation of sites on HIV’s glycoprotein gp120 results in enhanced immunogenicity and neutralising antibody production^14,34^. Interestingly, a fraction of HIV positive patients are capable of developing antibodies to the glycan shield itself^35^. Although, the SARS-CoV-2 S-protein is not as glycosylated as the HIV glycan shield, the glycan chains mask critical areas of the S-protein; hence the use of the term “glycan cloud”. It will be interesting to ascertain whether convalescent sera of SARS-CoV-2 individuals recognise the glycan cloud. The binding of recently identified SAR-CoVs antibodies S309 and BD-23 from convalescent patients have already been shown to be facilitated by the presence of the glycan chain^10,22^.

Ultimately, we provide a selection of B-cell epitopes that are exposed to the host immune system including epitopes located in flexible loops, which were previously overlooked. Some suggested epitopes remain exposed regardless of the type or conformation of glycan chains at N-glycosylation sites on the SARS-CoV-2 trimeric spike. The S-protein of the SARS-CoV-2 has proven to be highly conserved this far into the pandemic^36^, supporting the selection of epitopes on the S-protein. As monoclonal antibodies enter clinical trials, it is crucial to monitor the sequence variation in the S-protein for potential escape mutants. Variants anywhere in the S-protein have the potential to impact the 3D structure. Thus, genome sequence of the whole S-protein should be highly monitored. HIV escape mutants were found to evade the host antibodies without changing the epitope sequence, and rather by changing the glycan chain orientations at alternative sites, masking the previously exposed epitope^33^. This highlights the need for the epitope as well as the glycosylation pattern to be conserved.

In conclusion, we constructed a 3D model of the SARS-CoV-2 trimeric S-protein, completed with the flexible loops and N-linked glycan chains. This structure successfully enabled protruding epitopes, unmasked by the glycan cloud to be elucidated. Such epitopes can serve as targets for antibody therapeutics or to be incorporated in the urgently needed vaccines.

## METHODS

### Study design

This study used a structure-based approach of the SARS-CoV-2 Spike protein, taking into account the glycan chains, with the objective of identify B-cell epitopes unshielded by glycosylation. A complete trimeric 3D model of the S-protein was first built and used a based on which several glycosylated models were generated. As glycosylation is a dynamic process, six different carbohydrate chains were used, assuming complete and homogeneous glycosylation site occupancy. In addition, two heterogeneous glycosylation patterns were included (see Supplementary Table 4). Epitopes were identified based on accessible surface area calculations in absence or in presence of the different glycan chains.

### Reconstruction of missing residues in the trimeric spike

As initial model for our analysis we used a pre-fusion structure of the trimeric S-protein solved by cryo-EM at 2.80 Å resolution^14^ (PDB id 6VXX). Amino acids missing in the structure were constructed using Modeller9.24^37^. These residues are in the following loop segments: 70-79, 144-164, 173-185, 246-262, 445-446, 445-446, 469-488, 502, 621-640, 677-688 and 828-853. The modelling was performed in the presence of N-acetylglucosamine moieties found in the cryo-EM structure, with the exception of the Asn122 site whereby the N-attached N-acetylglucosamine has been replaced by a larger glycan (denoted here as “hybrid”, see Fig. 2c) in order to reduce the interatomic clashes between protein and glycan during the next step of *in silico* attachment of N-glycan. One hundred different models were generated by the loop modelling process. Note that no satisfactory modelling solution could be obtained for the largest loop 828-853. Using the five models with the lowest energies, geometries of the constructed loops were manually regularised in Coot^38^. The final models exhibit a suitable quality determined by MolProbity scores between 2.06-2.16^39^. Images of 3D model were produced with PyMOL (The PyMOL Molecular Graphics System, Version 2.3.0 Schrödinger, LLC).

### Building fully glycosylated trimer model of SARS-CoV-2 S-protein

The GlyProt tool (Glycosciences.DB portal^40^ (www.glycosciences.de)) was employed to attach carbohydrate chains at each N-linked glycosylation sites onto the full trimeric S-protein structure^41^. Six different glycans were considered for full homogeneously glycosylation patterns that are schematically depicted in Fig. 2b: three high-mannose type, one hybrid and two complex types (NAc(4)Man(3)Fuc(1)Gal(2)Neu(2) (Complex-12) and NAc(6)Man(3)Fuc(1)Gal(4)(Neu(3) (Complex-15)). In this study, it was assumed that all possible N-glycosylation sites were occupied. A further two patterns of N-glycosylation were considered based on the most frequent glycan found at each N-site of experimentally determined glycosylation data. The numbers of glycan residues besides the common structure of two N-acetylglucosamine moieties is also noted (see Fig. 2b). As glycans form a dynamic landscape on the surface of the protein, two different glycan conformations were modelled at each given glycosylation site. The first conformation extended the existing glycan in the orientation elucidated in the cryo-EM structure^14^, and the second conformation was built from a statistical evaluation of the occurrence of N-glycan orientations in the PDB^39^. Thus, ten models were proposed for each of the SARS-CoV-2 glycosylation patterns. All the 3D models are available from the authors upon request.

### Accessible surface area (ASA) calculations

In order to predict potential epitope exposed to the immune system unshielded by glycosylation, the ASA was computed for each model as well as de-glycosylated model using FreeSASA according to the Lee & Richards algorithm^17^. Two different probe radiuses were considered for each model during ASA calculations. Firstly, the solvent-ASA (SASA) was determined using a 1.4 Å radius to model a water molecule. Subsequently a 10 Å radius was use as this size is comparable to an antibody recognise region, resulting in the calculated antibody-ASA (AASA). To determine the AASA profiles, a smoothing procedure was applied consisting in a seven-point moving window procedure according the formula listed below, where N_i_ is the considered residue at position *i*.

N_*i*_ = [7N_*i*_ +6(N_*i*−1_ +N_*i*+1_)+3(N_*i*−2_ +N_*i*+2_)−2(N_*i*−3_ +N_*i*+3_)]/21^42^. The B-cell epitopes were determined using a cut-off of 45 Å^2^, chosen to produce a good concordance between our predictions and experimental epitopes derived from the structure between the SARS-CoV-2 S-protein and the neutralising monoclonal antibody S309^10^. All epitopes that were less than 3 residues in length were excluded. Epitopes separated by a single residue were grouped together. At one given position, the SASA or AASA was averaged over the three chains, over the five loop models and over the two models of glycan conformation. To quantify the impact of glycan cloud, the AASA is expressed as a percentage in relation to the value of the de-glycosylated model taken as 100%.

### Reporting summary

Further information on research design is available in the Nature Research Reporting Summary linked to this article.

## Supplementary information

Supplementary Information

Reporting Summary

## Data Availability

The datasets generated during and/or analysed during the current study are available from the corresponding author on reasonable request.
