# Supplementary material for: Impact of glycan cloud on the B-cell epitope prediction of SARS-CoV-2 Spike protein
Source: NPJ Vaccines. 2020 Sep 4;5:81. doi: 10.1038/s41541-020-00237-9 (PMC7474083; doi:10.1038/s41541-020-00237-9)
Supplement: Supplementary file 1 — Supplementary Information [file 41541_2020_237_MOESM1_ESM.pdf]

Supplementary Materials

Impact of glycan cloud on the B-cell epitope prediction of SARS-CoV-2 Spike protein

René Wintjens[1*], Amanda Makha Bifani[2], Pablo Bifani[3,4,5*]

[1]Unit of Microbiology, Bioorganic and Macromolecular Chemistry, Department of Research in Drug Development (RD3), Faculté de Pharmacie, Université Libre de Bruxelles, 1050 Brussels, Belgium.

[2]Programme in Emerging Infectious Diseases, Duke-NUS Medical School, 169857, Singapore.

[3]Singapore Immunology Network (SIgN), A*STAR, 8A Biomedical Grove, Immunos Building, Singapore 138648, Singapore.

[4]Infectious Diseases Programme and Department of Microbiology and Immunology, Yong Loo Lin School of Medicine, National University of Singapore, 119077 Singapore.

[5]Department of Infection Biology, Faculty of Infectious and Tropical Diseases, London School of Hygiene and Tropical Medicine, London WC1E 7HT, United Kingdom.

[*]Correspondence: René Wintjens: rene.wintjens@ulb.ac.be; Pablo Bifani: micpb@nus.edu.sg

Supplementary Table 1. AASA (Å^2) of structural domains as a function of glycan chains.

	no-gly	CryoEM	Man3	Man5	Pat1	Pat2	Man9	Hyb8	Com10	Com15
**PROT**	31802.31	25088.41	16859.25	15536.98	14138.63	15790.59	14236.98	12911.53	10067.62	6578.38
	100.0%	78.9%	53.0%	48.9%	44.5%	49.7%	44.8%	40.6%	31.7%	20.7%
**Glycan**	0	7672.09	22066.20	23562.80	26653.40	26262.20	26492.30	32824.50	31341.90	46254.80
**NTD**	10820.2	10018.12	6597.82	6149.9	5733.11	6236.17	5709.84	5426.98	4050.01	3129.3
	100.0%	92.6%	61.0%	56.8%	53.0%	57.6%	52.8%	50.2%	37.4%	28.9%
**RBD**	5930.15	5325.22	4001.66	3825.79	3200.02	3840.81	3442.73	2952.03	2731.38	1677.15
	100.0%	89.8%	67.5%	64.5%	54.0%	64.8%	58.1%	49.8%	46.1%	28.3%
**SD1**	1929.66	1385.96	686.44	572.44	498.64	590.22	482.64	430.95	229.34	85.34
	100.0%	71.8%	35.6%	29.7%	25.8%	30.6%	25.0%	22.3%	11.9%	4.4%
**SD2**	3544.45	2494.11	1754.53	1648.81	1590.84	1860.59	1344.96	989.7	1198.68	525.71
	100.0%	70.4%	49.5%	46.5%	44.9%	52.5%	37.9%	27.9%	33.8%	14.8%
**CL**	2637.86	2248.28	1854.09	1791.83	1798.24	1666.15	1733.79	1623.42	1380.86	796.55
	100.0%	85.2%	70.3%	67.9%	68.2%	63.2%	65.7%	61.5%	52.3%	30.2%
**UH**	0.04	0	0	0	0	0	0	0	0	0
	100.0%	0.0%	0.0%	0.0%	0.0%	0.0%	0.0%	0.0%	0.0%	0.0%
**FP**	318.18	184.09	48.1	33.68	25.27	133.2	61.84	71.57	0	0
	100.0%	57.9%	15.1%	10.6%	7.9%	41.9%	19.4%	22.5%	0.0%	0.0%
**CR**	15.54	10.61	1.42	1.36	1.49	2.67	1.15	1.73	0	0
	100.0%	68.3%	9.1%	8.8%	9.6%	17.2%	7.4%	11.1%	0.0%	0.0%
**HR1**	1132.67	567.36	219.37	175.15	148.06	181.12	196.94	221.46	40	9.64
	100.0%	50.1%	19.4%	15.5%	13.1%	16.0%	17.4%	19.6%	3.5%	0.9%
**CH**	0	0	0	0	0	0	0	0	0	0
**BH**	286.83	92.82	16.91	20.32	19.01	2.7	19.46	58.88	10.03	0
	100.0%	32.4%	5.9%	7.1%	6.6%	0.9%	6.8%	20.5%	3.5%	0.0%
**SD3**	2433.6	671.85	372.4	233.7	197.5	307.04	172.55	205.1	79.03	1.94
	100.0%	27.6%	15.3%	9.6%	8.1%	12.6%	7.1%	8.4%	3.2%	0.1%

Supplementary Table 1. AASA (A^2) of structural domains as a function of glycan chains. Glycan chains size and type diminish the antibody accessible surface area (AASA) of the SARS-CoV-2 S-protein. Table showing the AASA along the structural domains of the S-protein using different glycan chains species. The accessible surface area is depicted for each structural domain with the percentage of the accessible surface listed underneath.

Supplementary Table 2. Distance matrix between the 28 predicted B-epitopes

#1	#2	#3	#4	#5	#6	#7	#8	#9	#10	#11	#12	#13	#14	#15	#16	#17	#18	#19	#20	#21	#22	#23	#24	#25	#26	#27	#28	
0	17	32	34	20	22	50	61	70	87	113	80	51	64	63	50	42	60	51	96	83	93	71	90	121	106	116	113	#1
	0	22	36	30	29	50	53	58	73	101	65	48	63	59	56	49	68	62	101	87	101	77	98	128	115	122	121	#2
		0	39	42	28	59	71	71	83	112	73	68	84	79	70	69	87	79	111	95	113	90	114	145	131	140	137	#3
			0	21	31	25	75	79	92	114	83	65	74	76	37	54	61	52	73	58	76	54	80	112	98	110	105	#4
				0	28	34	65	75	91	114	84	53	62	64	32	37	48	38	77	64	75	52	73	105	90	102	97	#5
					0	55	80	86	101	128	92	71	84	83	60	60	75	64	101	87	100	79	101	133	117	129	124	#6
						0	69	73	85	101	77	60	62	67	23	51	49	46	53	37	58	35	62	93	81	90	86	#7
							0	21	41	61	41	17	29	16	68	51	66	72	108	98	105	83	95	116	110	107	112	#8
								0	20	46	19	37	47	36	78	68	82	88	115	104	115	93	109	129	124	120	126	#9
									0	33	11	56	64	54	93	87	99	106	126	114	128	106	123	142	138	133	139	#10
										0	41	74	74	68	107	105	110	121	131	122	134	116	128	141	142	131	140	#11
											0	54	64	54	87	83	95	101	121	108	124	101	120	141	136	132	137	#12
												0	18	11	54	34	49	56	96	87	91	70	81	103	95	94	98	#13
													0	13	52	36	42	53	89	82	82	64	69	88	82	78	84	#14
														0	61	42	53	62	100	92	94	74	83	102	96	92	98	#15
															0	36	27	25	48	37	45	21	44	75	62	72	68	#16
																0	28	28	81	73	72	52	61	88	75	81	80	#17
																	0	18	59	56	48	32	34	62	48	56	54	#18
																		0	62	56	51	35	42	72	56	69	64	#19
																			0	17	19	28	38	57	50	61	53	#20
																				0	29	23	45	70	61	72	64	#21
																					0	24	21	43	32	47	37	#22
																						0	28	59	46	57	51	#23
																							0	32	18	32	24	#24
																								0	20	12	9	#25
																									0	23	10	#26
																										0	15	#27
																											0	#28

Legend. The distances (in Å) were calculated between the α carbon of the central residue of each epitope. From epi1 to epi28, the central residues are N74, N137, E154, E180, R214, D253, E281, T333, T345, G447, N481, P499, N532, S555, T581, N603, P631, N657, R682, I794, S810, K921, E1072, D1084, T1100, D1127 and N1135, respectively. The distances were averaged over the three chains and over the 5 models. Considering the size of an antibody paratope (i.e. the six CDR loops), two epitopes with a distance less than ~25 Å can be recognized by a single antibody. A color scale for the distances is added in the table.

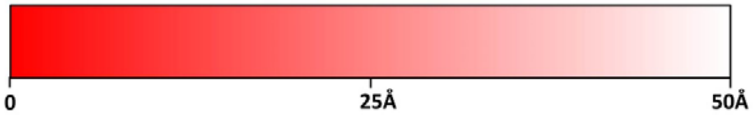

Supplementary Table 2. Distance matrix between the 28 predicted B-epitopes. The distances (in Å) were calculated between the α carbon of the central residue of each epitope. From epi1 to epi28, the central residues are N74, N137, E154, E180, R214, D253, E281, T333, T345, G447, N481, P499, N532, S555, T581, N603, P631, N657, R682, I794, S810, K921, E1072, D1084, T1100, D1127 and N1135, respectively. The distances were averaged over the three chains and the 5 models. Considering the size of an antibody paratope (i.e. the six CDR loops), two epitopes with a distance less than ~25 Å can be recognized by a single antibody. A colour scale for the distances is added in the table.

Supplementary Table 3. Concordance between the predicted epitopes and antibody binding sites reported in the literature.

Antibody	Domain	Experimental binding site	predicted epitope	Reference	comment
4A8	NTD	141-156, 246-260	epi3-epi6	Chi et al. DOI: 10.1101/2020.05.08.083964	
S309	RBD	333-346, 354-361, 440-441	epi9-epi10	Pinto et al, Ref #10	354-361 not found
47D11	RBD	438-498	epi10	Wang et al. DOI: 10.1038/s41467-020-16256-y	
CR3022	RBD	369-392, 427-430, 515-519	FAILED	Yuan et al. DOI: 10.1126/science.abb7269	non-neutralizing antibody
B38	RBD	403-421, 449-460, 473-505	epi10 to epi12	Wu et al. DOI: 10.1126/science.abc2241	403-421 not found
BD23	RBD	446-456, 470-505	epi10 to epi12	Cao et al. Ref #22	
COV2-2130	RBD	K444, G447	epi10	Zost et al. Ref #23	partially defined binding site
COV2-2165	RBD	N487	epi11	Zost et al. Ref #23	partially defined binding site
COV2-2196	RBD	F486, N487	epi11	Zost et al. Ref #23	partially defined binding site
S14P5	SD1	553-570	epi14	Poh et al. Ref #24	
S21P2	FP	809-826	epi21	Poh et al. Ref #24	
1A9	SD3	1129-1148	epi28	Zheng et al. DOI: 10.1101/2020.03.06.980037	

Supplementary Table 3. Concordance between the predicted epitopes and antibody binding sites reported in the literature. Table highlighting the agreement in predicted epitopes from this study compared to previously experimentally determined antibody binding sites.

Supplementary Table 4. Glycosylation patterns of SARS-CoV-2 Spike protein based on experimental results

Feature	Watanabe et al 2020, Science	Shajahan et al 2020 , Glycobiology	Zhang et al 2020, bioRxiv 2020.03.28.013276
**Protein production**	Recombinantly expressed in human embryonic kidney 293F cells	HEK293 cells expressing subunits S1 and S2 separately	Human HEK293 (S1) and insect (full) cell-expressed protein (here below only results for S1 subunit expressed in human cells)
**Experimental procedure**	Trypsin, chymotrypsin and alpha-lytic protease treatment followed by LC-MS analysis	Trypsin and chymotrypsin digestion followed LC-MS/MS analysis	Trypsin and Glu-C digestion followed by LC-MS/MS analysis
**Number of N-glycosylation sites**	22 (ALL)	17	22 (ALL)
**O-linked glycosylation**	Trace levels (<1%) at T323/S325	Two O-glycosylation sites T323/S325	not analyzed
**Glycan compositions**	28% mannose, 5% hybrid, 66% complex	percentages not given	~12% mannose, ~13% hybrid, ~75% complex
**Top glycan at N17**	NAc(5)Man(3)Fuc(1)Gal(1)	non-glycosylated	complex
**Top glycan at N61**	NAc(2)Man(5)	NAc(2)Man(5)	complex
**Top glycan at N74**	NAc(5)Man(3)Fuc(1)Gal(1)	NAc(4)Man(3)Fuc(1)	complex
**Top glycan at N122**	NAc(2)Man(5)	NAc(2)Man(5)	complex
**Top glycan at N149**	NAc(4)Man(3)Fuc(1)	NAc(5)Man(3)	complex
**Top glycan at N165**	NAc(5)Man(4)Fuc(1)Gal(1)	NAc(3)Man(3)Gal(1)	complex
**Top glycan at N234**	NAc(2)Man(8)	NAc(2)Man(5)	complex
**Top glycan at N282**	NAc(5)Man(3)Fuc(1)	NAc(5)Man(3)Fuc(1)	complex
**Top glycan at N331**	NAc(4)Man(3)Fuc(1)	NAc(2)Man(5)	complex
**Top glycan at N343**	NAc(4)Man(3)Fuc(1)Gal(2)	NAc(2)Man(5)	complex
**Top glycan at N603**	NAc(2)Man(5)	non-glycosylated	high-mannose
**Top glycan at N616**	NAc(4)Man(3)Fuc(1)	NAc(2)Man(5)	complex
**Top glycan at N657**	NAc(4)Man(3)Fuc(1)	NAc(4)Man(3)Fuc(1)Gal(2)	complex
**Top glycan at**	NAc(2)Man(5)	NAc(4)Man(3)Gal(1)Neu(1)	not determined

**N709**			
**Top glycan at N717**	NAc(2)Man(5)	NAc(2)Man(5)	not determined
**Top glycan at N801**	NAc(2)Man(5)	NAc(4)Man(3)Gal(2)Fuc(1)Neu(1)	not determined
**Top glycan N1074**	NAc(2)Man(5)	NAc(6)Man(3)Gal(4)Fuc(1)Neu(1)	not determined
**Top glycan N1098**	NAc(3)Man(4)Gal(1)	NAc(6)Man(3)Gal(4)Neu(2)	not determined
**Top glycan N1134**	NAc(4)Man(3)Fuc(1)	non-glycosylated	not determined
**Top glycan N1158**	NAc(4)Man(3)Gal(1)	non-glycosylated	not determined
**Top glycan N1173**	NAc(6)Man(3)Fuc(1)	non-glycosylated	not determined
**Top glycan N1194**	NAc(6)Man(3)Fuc(1)Gal(2)Neu(1)	NAc(6)Man(3)Gal(4)Fuc(1)Neu(4)	not determined

High-mannose type
hybrid type glycan
complex type glycan

Supplementary Table 4. Glycosylation patterns of SARS-CoV-2 Spike protein based on experimental results. Table comparing glycosylation patterns observed in HEK and insect cells across three different previously published papers (Shajahan, et. al., Glycobiology, doi:10.1093/glycob/cwaa042 (2020); Watanabe, et. al., Science, doi:10.1126/science.abb9983 (2020); Zhang, Y. et. al., bioRxiv, doi:10.1101/2020.03.28.013276 (2020)).

Supplementary Table 5. SASA (Å2) of structural domains as a function of glycan chains.

	no-gly	CryoEM	Man3	Man5	Pat1	Pat2	Man9	Hyb8	Com10	Com15
**PROT**	50972.89	49103.3	48508.57	47957.25	47232.78	47893.67	47101.07	47296.59	46097.46	45033.66
	100.0%	96.3%	95.2%	94.1%	92.7%	94.0%	92.4%	92.8%	90.4%	88.3%
**Glycan**	0.00	5014.88	15483.90	18079.20	22518.80	22625.80	23920.80	30884.00	36773.40	55798.70
**NTD**	15000.64	14513.48	14226.62	14059.65	13906.86	14003.66	13875.38	14026.4	13464.66	13281.38
	100.0%	96.8%	94.8%	93.7%	92.7%	93.4%	92.5%	93.5%	89.8%	88.5%
**RBD**	9910.3	9622.86	9399.47	9329.58	8931.19	9240.97	9089.51	9119.24	8943.89	8310.15
	100.0%	97.1%	94.8%	94.1%	90.1%	93.2%	91.7%	92.0%	90.2%	83.9%
**SD1**	3018.81	2962.15	2986.79	2977.47	2914.77	2941.4	2951.49	2901.46	2884.84	2892.36
	100.0%	98.1%	98.9%	98.6%	96.6%	97.4%	97.8%	96.1%	95.6%	95.8%
**SD2**	6226.37	5937.3	5901.07	5848.72	5760.76	5931.46	5693.45	5664.29	5652.31	5496.33
	100.0%	95.4%	94.8%	93.9%	92.5%	95.3%	91.4%	91.0%	90.8%	88.3%
**CL**	2869.28	2699.92	2640.37	2572.35	2573.29	2583.23	2544.44	2535.92	2476.48	2500.93
	100.0%	94.1%	92.0%	89.7%	89.7%	90.0%	88.7%	88.4%	86.3%	87.2%
**UH**	1504.36	1504.36	1504.36	1504.36	1504.36	1504.36	1504.36	1504.34	1497.92	1490.51
	100.0%	100.0%	100.0%	100.0%	100.0%	100.0%	100.0%	100.0%	99.6%	99.1%
**FP**	504.79	498.5	446.05	462.08	462.08	493.51	386.73	411.76	466.3	380.06
	100.0%	98.8%	88.4%	91.5%	91.5%	97.8%	76.6%	81.6%	92.4%	75.3%
**CR**	1283.27	1282.93	1285.25	1285.3	1285.3	1284.05	1280.64	1250.2	1284.53	1258.32
	100.0%	100.0%	100.2%	100.2%	100.2%	100.1%	99.8%	97.4%	100.1%	98.1%
**HR1**	2738.07	2646.51	2596.68	2507.53	2507.53	2515.91	2458.57	2478.6	2430.56	2423.09
	100.0%	96.7%	94.8%	91.6%	91.6%	91.9%	89.8%	90.5%	88.8%	88.5%
**CH**	1654.48	1654.48	1654.48	1654.48	1654.48	1654.48	1654.48	1654.48	1648.25	1638.71
	100.0%	100.0%	100.0%	100.0%	100.0%	100.0%	100.0%	100.0%	99.6%	99.0%
**BH**	464.91	455.85	443.68	445.01	445.01	445.01	439.66	410.62	445.11	439.91
	100.0%	98.1%	95.4%	95.7%	95.7%	95.7%	94.6%	88.3%	95.7%	94.6%
**SD3**	3029.38	2682.97	2750.97	2695.77	2685.57	2758.73	2666.46	2707.03	2494.31	2620.22
	100.0%	88.6%	90.8%	89.0%	88.7%	91.1%	88.0%	89.4%	82.3%	86.5%

Supplementary Table 5. SASA of structural domains as a function of glycan chains. Glycan chains have a limited effect on the solvent accessible surface area (SASA) of the SARS-CoV-2 S-protein. Table showing the SASA for a solvent equal to the size of a H2O molecule along the structural domains of the S-protein using different glycan chains species. The total accessible surface area is provided with the percentage of the accessible surface depicted below.

Figure S1

```
                                 17▼17
                   SP                   NTD
SARS-CoV2    1-----MFVFLVLLPLVSSQCVNLTTRTQLPPAYTNSFTRGVYYPDKVFRSSVLHSTQDLFL-
56
         DSSP:                               EEE           EE   EEEEEEEEE

                   ▼61          ▼74
                                   NTD
SARS-CoV2   57-PFFSNVTWFHAIHVSGTNGTKRFDNPVLPFNDGVYFASTEKSNIIRGWIFGTTLDSKTQS-
116
         DSSP:    EEEEE                 EEE    EEEEEEE    EEEEEEE

                    ▼122                        ▼149            ▼165
                                  NTD
SARS-CoV2  117-LLIVNNATNVVIKVCEFQFCNDPFLGVYYHKNNKSWMESEFRVYSSANNCTFEYVSQPFL-
176
         DSSP: EEEEEE  EEEEE           EE                          EEE

                                                                     ▼234
                                  NTD
SARS-CoV2  177-MDLEGKQGNFKNLREFVFKNIDGYFKIYSKHTPINLVRDLPQGFSALEPLVDLPIGINIT-
236
         DSSP:            EEEEEEEEEE  EEEEEEEEEE              EEEEEEE

                                                              ▼282
                                  NTD
SARS-CoV2  237-RFQTLLALHRSYLTPGDSSSGWTAGAAAYYVGYLQPRTFLLKYNENGTITDAVDCALDPL-
296
         DSSP: EEEEEEEE                    EEEEE EEEEEEEEE     EEEEEE    HH

                      305              322      330▼331        ▼343
                 NTD         SD2               SD1         RBD
SARS-CoV2  297-SETKCTLKSFTVEKGIYQTSNFRVQPTESIVRFPNITNLCPFGEVFNATRFASVYAWNRK-
356
         DSSP: HHHHHHH       EEEEEEEEE     EEEE          HHHH       GGG EEE

                                   RBD
SARS-CoV2  357-RISNCVADYSVLYNSASFSTFKCYGVSPTKLNDLCFTNVYADSFVIRGDEVRQIAPGQTG-
416
         DSSP: EE  EE  HHHHHH     EEEE              EEEEEEEEEEGGGGGG

                                   RBD
SARS-CoV2  417-KIADYNYKLPDDFTGCVIAWNSNNLDSKVGGNYNYLYRLFRKSNLKPFERDISTEIYQAG-
476
         DSSP:              EEEEEEE          EEEEEE

                                                                527
                            RBD                                     SD1
SARS-CoV2  477-STPCNGVEGFNCYFPLQSYGFQPTNGVGYQPYRVVVLSFELLHAPATVCGPKKSTNLVKN-
536
         DSSP:                EEEEE         EEEEEEEEEE        EE

                                                                   590
                               SD1                                    SD2
```

```
SARS-CoV2   537-KCVNFNFNGLTGTGVLTESNKKFLPFQQFGRDIADTTDAVRDPQTLEILDITPCSFGGVS-
596
        DSSP:  EEEEEE  EEEEEEEEE          EEE     EEEEE      EEEEE   EEEEE

                  ▼603         ▼616
                                 SD2
SARS-CoV2   597-VITPGTNTSNQVAVLYQDVNCTEVPVAIHADQLTPTWRVYSTGSNVFQTRAGCLIGAEHV-
656
        DSSP: EEE         EEEEE                                EEEE  EEEE  EE

                ▼657               676     S1/S2 689     698      709▼709  716
                       SD2                   CL          SD2        L      SD3
SARS-CoV2   657-NNSYECDIPIGAGICASYQTQTNSPRRARSVASQSIIAYTMSLGAENSVAYSNNSIAIPT-
716
        DSSP:        EEEE  EEEEEE                    EEEEEE    EE         EEEEEE

                ▼717         730    737
                       BH        CR             UH
SARS-CoV2   717-NFTISVTTEILPVSMTKTSVDCTMYICGDSTECSNLLLQYGSFCTQLNRALTGIAVEQDK-
776
        DSSP: EEEEEEEEEEEE    EEEE HHHHH    HHHHHHGGGGG HHHHHHHHHHHHHHHH

                     783               ▼801         815         827
                  UH              L                      FP        L
SARS-CoV2   777-NTQEVFAQVKQIYKTPPIKDFGGFNFSQILPDPSKPSKRSFIEDLLFNKVTLADAGFIKQ-
836
        DSSP: HHHHHH    EE                             HHHHHHH

                  840
                                     CR
SARS-CoV2   837-YGDCLGDIAARDLICAQKFNGLTVLPPLLTDEMIAQYTSALLAGTITSGWTFGAGAALQI-
896
        DSSP:                      EEEE     HHHHHHHHHHHHHHHHHH  HHHH

                             911
                  CR                        HR1
SARS-CoV2   897-PFAMQMAYRFNGIGVTQNVLYENQKLIANQFNSAIGKIQDSLSSTASALGKLQDVVNQNA-
956
        DSSP:  HHHHHHHHHH      HHHHH HHHHHHHHHHHHHHHHHHHHH GGGHHHHHHHHHHHH

                                         984
                        HR1                             CH
SARS-CoV2   957-QALNTLVKQLSSNFGAISSVLNDILSRLDKVEAEVQIDRLITGRLQSLQTYVTQQLIRAA-
1016
        DSSP: HHHHHHHHGGG         HHHHH    HHHHHHHHHHHHHHHHHHHHHHHHHHHHHHH

                                     1043                        1071 ▼1074
                         CH                         BH
SARS-CoV2 1017-EIRASANLAATKMSECVLGQSKRVDFCGKGYHLMSFPQSAPHGVVFLHVTYVPAQEKNFT-
1076
        DSSP: HHHHHHHHHHHHHIIIII            EEEEEEEEEE  EEEEEEEEEEEEEEEEEE

                                ▼1098                  1122      ▼1134
                        SD3                                  L
SARS-CoV2 1077-TAPAICHDGKAHFPREGVFVSNGTHWFVTQRNFYEPQIITTDNTFVSGNCDVVIGIVNNT-
1136
```

```
          DSSP: EE   EE   EEEEE     EEEE               EE      EEEEEE        EE

                                         1158▼ 1162           ▼1173                   ▼1194
                          L                               HR2
SARS-CoV2 1137-VYDPLQPELDSFKEELDKYFKNHTSPDVDLGDISGINASVVNIQKEIDRLNEVAKNLNES-
1196
          DSSP:      HHHHH

                  1202        1213                   1234
                             L           TM                       CP
SARS-CoV2 1197-LIDLQELGKYEQYIKWPWYIWLGFIAGLIAIVMVTIMLCCMTSCCSCLKGCCSCGSCCKF-
1256

                       CP
SARS-CoV2 1257-DEDDSEPVLKGVKLHYT-1273
```

Supplementary Fig. 1. Structural features of the SARS-CoV-2 Spike protein. The sequence of the SARS-CoV-2 Spike protein is depicted with the different structural domains showed above and colored according the Figure 1a. The structural domains are: SP: signal peptide, NTD: N-terminal domain, L: linker region, RBD: receptor-binding domain, SD: subdomain, CL, cleavage loop, UH: upstream helix,FP: fusion peptide, CR: connecting region, HR: heptad repeat, CH: central helix, BH: β-hairpin, TM: transmembrane region, CP: cytoplasmic part. The secondary structures calculated with DSSP program are indicated below the sequence by letter 'G'= 3_{10} helix (3-turn helix), 'H'= α-helix (4-turn helix), 'I'= π-helix (5-turn helix) and 'E'= extended strand in parallel and/or antiparallel β-sheet conformation. The ending limit of each structural domain is adapted from Yuan et al., (2017; DOI: 10.1038/ncomms15092), and adjusted according β-sheet pairing and disulfide bridges (i.e. disulfide bridges have to involve two cysteines in the same domain). The cleavage site S1/S2 is showed by an arrow (i.e. "↓"). The N-linked glycosylation site is labeled and showed by ▼. Missing residues in the cryo-EM structure 6VXX are in grey. In each protomer, the 12 disulfide bridges are: C131-C166, C291-C301, C336-C361, C379-C432, C391-C525, C538-C590, C617-C649, C662-C671, C738-C760, C743-C749, C1032-C1043 and C1082-C1126.

Figure S2

```
                17          ▼29
SP             |           NTD
SARS-CoV    1-MFIFLLFLTLTSGSDLDRCTTFDDVQAPNYTQHTSSMRGVYYPDEIFRSDTLYLTQDLFL-
60
SARS-CoV2   1-MFVFLVLLPLVSSQCVN--LTTRTQLPPAYTN--SFTRGVYYPDKVFRSSVLHSTQDLFL-
56
              17▲17

      ▼65      ▼73                                   ▼109
                         NTD
SARS-CoV   61-PFYSNVTGFHTINHT-------FGNPVIPFKDGIYFAATEKSNVVRGWVFGSTMNNKSQS-
113
SARS-CoV2  57-PFFSNVTWFHAIHVSGTNGTKRFDNPVLPFNDGVYFASTEKSNIIRGWIFGTTLDSKTQS-
116
      ▲61         ▲74

   118▼▼119                                      ▼158
                         NTD
SARS-CoV  114-VIIINNSTNVVIRACNFELCDNPFFAVSKPMG----TQTHTMIFDNAFNCTFEYISDAFS-
169
SARS-CoV2 117-LLIVNNATNVVIKVCEFQFCNDPFLGVYYHKNNKSWMESEFRVYSSANNCTFEYVSQPFL-
176
        ▲122                      ▲149          ▲165

                                                       ▼227
                         NTD
SARS-CoV  170-LDVSEKSGNFKHLREFVFKNKDGFLYVYKGYQPIDVVRDLPSGFNTLKPIFKLPLGINIT-
229
SARS-CoV2 177-MDLEGKQGNFKNLREFVFKNIDGYFKIYSKHTPINLVRDLPQGFSALEPLVDLPIGINIT-
236
                                                       ▲234

                                              ▼269
                         NTD
SARS-CoV  230-NFRAILTAFS------PAQDIWGTSAAAYFVGYLKPTTFMLKYDENGTITDAVDCSQNPL-
283
SARS-CoV2 237-RFQTLLALHRSYLTPGDSSSGWTAGAAAYYVGYLQPRTFLLKYNENGTITDAVDCALDPL-
296
                                              ▲282

          292              299     317▼318          ▼330
NTD      |     SD2        |  SD1  |         RBD
SARS-CoV  284-AELKCSVKSFEIDKGIYQTSNFRVVPSGDVVRFPNITNLCPFGEVFNATKFPSVYAWERK-
343
SARS-CoV2 297-SETKCTLKSFTVEKGIYQTSNFRVQPTESIVRFPNITNLCPFGEVFNATRFASVYAWNRK-
356
          305              322     330▲331          ▲343

                ▼357
                         RBD
SARS-CoV  344-KISNCVADYSVLYNSTFFSTFKCYGVSATKLNDLCFSNVYADSFVVKGDDVRQIAPGQTG-
```

403

SARS-CoV2 357-RISNCVADYSVLYNSAFSTFKCYGVSPTKLNDLCFTNVYADSFVIRGDEVRQIAPGQTG-

416

RBD

SARS-CoV 404-VIADYNYKLPDDFMGCVLAWNTRNIDATSTGNYNYKYRYLRHGKLRPFERDISNVPFSPD-

463

SARS-CoV2 417-KIADYNYKLPDDFTGCVIAWNSNNLDSKVGGNYNYLYRLFRKSNLKPFERDISTEIYQAG-

476

513

RBD SD1

SARS-CoV 464-GKPCT-PPALNCYWPLNDYGFYTTTGIGYQPYRVVVLSFELLNAPATVCGPKLSTDLIKN-

522

SARS-CoV2 477-STPCNGVEGFNCYFPLQSYGFQPTNGVGYQPYRVVVLSFELLHAPATVCGPKKSTNLVKN-

536

527

575

SD1 SD2

SARS-CoV 523-QCVNFNFNGLTGTGVLTPSSKRFQPFQQFGRDVSDFTDSVRDPKTSEILDISPCSFGGVS-

582

SARS-CoV2 537-KCVNFNFNGLTGTGVLTESNKKFLPFQQFGRDIADTTDAVRDPQTLEILDITPCSFGGVS-

596

589

▼589 ▼602

SD2

SARS-CoV 583-VITPGTNASSEVAVLYQDVNCTDVSTAIHADQLTPAWRIYSTGNNVFQTQAGCLIGAEHV-

642

SARS-CoV2 597-VITPGTNTSNQVAVLYQDVNCTEVPVAIHADQLTPTWRVYSTGSNVFQTRAGCLIGAEHV-

656

▲603 ▲616

662 S1/S2 672 680 ▼691

SD2 CL ↓ SD2 L

SARS-CoV 643-DTSYECDIPIGAGICASYHTVS----LLRSTSQKSIVAYTMSLGADSSIAYSNNTIAIPT-

698

SARS-CoV2 657-NNSYECDIPIGAGICASYQTQTNSPRRARSVASQSIIAYTMSLGAENSVAYSNNSIAIPT-

716

↑

▲657 676 S1/S2 689 698 ▲709

▼699 712 719

BH UH

SARS-CoV 699-NFSISITTEVMPVSMAKTSVDCNMYICGDSTECANLLLQYGSFCTQLNRALSGIAAEQDR-

758

SARS-CoV2 717-NFTISVTTEILPVSMTKTSVDCTMYICGDSTECSNLLLQYGSFCTQLNRALTGIAVEQDK-

776

```
                 ▲717          730     737

                       765                    ▼783               797          809
                 UH     |        L             |                  |     FP     |    L
SARS-CoV    759-NTREVFAQVKQMYKTPTLKYFGGFNFSQILPDPLKPTKRSFIEDLLFNKVTLADAGFMKQ-
818
SARS-CoV2   777-NTQEVFAQVKQIYKTPPIKDFGGFNFSQILPDPSKPSKRSFIEDLLFNKVTLADAGFIKQ-
836
                        |                      |                  |            |
                       783                    ▲801               815          827

                    822
                        |                          CR
SARS-CoV    819-YGECLGDINARDLICAQKFNGLTVLPPLLTDDMIAAYTAALVSGTATAGWTFGAGAALQI-
878
SARS-CoV2   837-YGDCLGDIAARDLICAQKFNGLTVLPPLLTDEMIAQYTSALLAGTITSGWTFGAGAALQI-
896
                        |
                    840

                                893
                  CR             |                   HR1
SARS-CoV    879-PFAMQMAYRFNGIGVTQNVLYENQKQIANQFNKAISQIQESLTTTSTALGKLQDVVNQNA-
938
SARS-CoV2   897-PFAMQMAYRFNGIGVTQNVLYENQKLIANQFNSAIGKIQDSLSSTASALGKLQDVVNQNA-
956
                                 |
                                911

                                             966
                       HR1                    |                CH
SARS-CoV    939-QALNTLVKQLSSNFGAISSVLNDILSRLDKVEAEVQIDRLITGRLQSLQTYVTQQLIRAA-
998
SARS-CoV2   957-QALNTLVKQLSSNFGAISSVLNDILSRLDKVEAEVQIDRLITGRLQSLQTYVTQQLIRAA-
1016
                                              |
                                             984

                                 1016                                      1053 ▼1056
                  CH               |                  BH                     |   |
SARS-CoV    999-EIRASANLAATKMSECVLGQSKRVDFCGKGYHLMSFPQAAPHGVVFLHVTYVPSQERNFT-
1058
SARS-CoV2  1017-EIRASANLAATKMSECVLGQSKRVDFCGKGYHLMSFPQSAPHGVVFLHVTYVPAQEKNFT-
1076
                                   |                                         |   |
                                 1034                                      1071 ▲1074
                          ▼1080                             1104                ▼1116
                       SD3  |                                 |        L         |
SARS-CoV   1059-TAPAICHEGKAYFPREGVFVFNGTSWFITQRNFFSPQIITTDNTFVSGNCDVVIGIINNT-
1118
SARS-CoV2  1077-TAPAICHDGKAHFPREGVFVSNGTHWFVTQRNFYEPQIITTDNTFVSGNCDVVIGIVNNT-
1136
                            |                                 |                  |
                          ▲1098                              1122               ▲1134

                        1140▼  1144         ▼1155                               ▼1176
                 L          |      |           |       HR2                       |
```

```
SARS-CoV  1119-VYDPLQPELDSFKEELDKYFKNHTSPDVDLGDISGINASVVNIQKEIDRLNEVAKNLNES-
1178
SARS-CoV2 1137-VYDPLQPELDSFKEELDKYFKNHTSPDVDLGDISGINASVVNIQKEIDRLNEVAKNLNES-
1196

                         1158▲  1162          ▲1173                    ▲1194


                  1184        1195                    1216
                          L             TM                     CP
SARS-CoV  1179-LIDLQELGKYEQYIKWPWYVWLGFIAGLIAIVMVTILLCCMTSCCSCLKGACSCGSCCKF-
1238
SARS-CoV2 1197-LIDLQELGKYEQYIKWPWYIWLGFIAGLIAIVMVTIMLCCMTSCCSCLKGCCSCGSCCKF-
1256

                  1202        1213                    1234


                CP
SARS-CoV  1239-DEDDSEPVLKGVKLHYT-1255
SARS-CoV2 1257-DEDDSEPVLKGVKLHYT-1273
```

Supplementary Fig. 2. SARS-CoV and SARS-CoV-2 subunits S2 share similar N-glycosylation profiles, while the S1 subunits are more distinct between the two species. Sequence alignment of SARS-CoV and SARS-CoV2 primary structures with the different structural domains colored according the Figure 1a. SP: signal peptide, NTD: N-terminal domain, L: linker region, RBD: receptor-binding domain, SD: subdomain, CL, cleavage loop, UH: upstream helix,FP: fusion peptide, CR: connecting region, HR: heptad repeat, CH: central helix, BH: β-hairpin, TM: transmembrane region, CP: cytoplasmic part. The ending limit of each structural domain is indicated. The structural domains are defined as indicated in the legend of Fig. S1. The cleavage site S1/S2 is showed by an arrow (i.e. "↑"). The N-linked glycosylation site is labeled and showed by ▼.

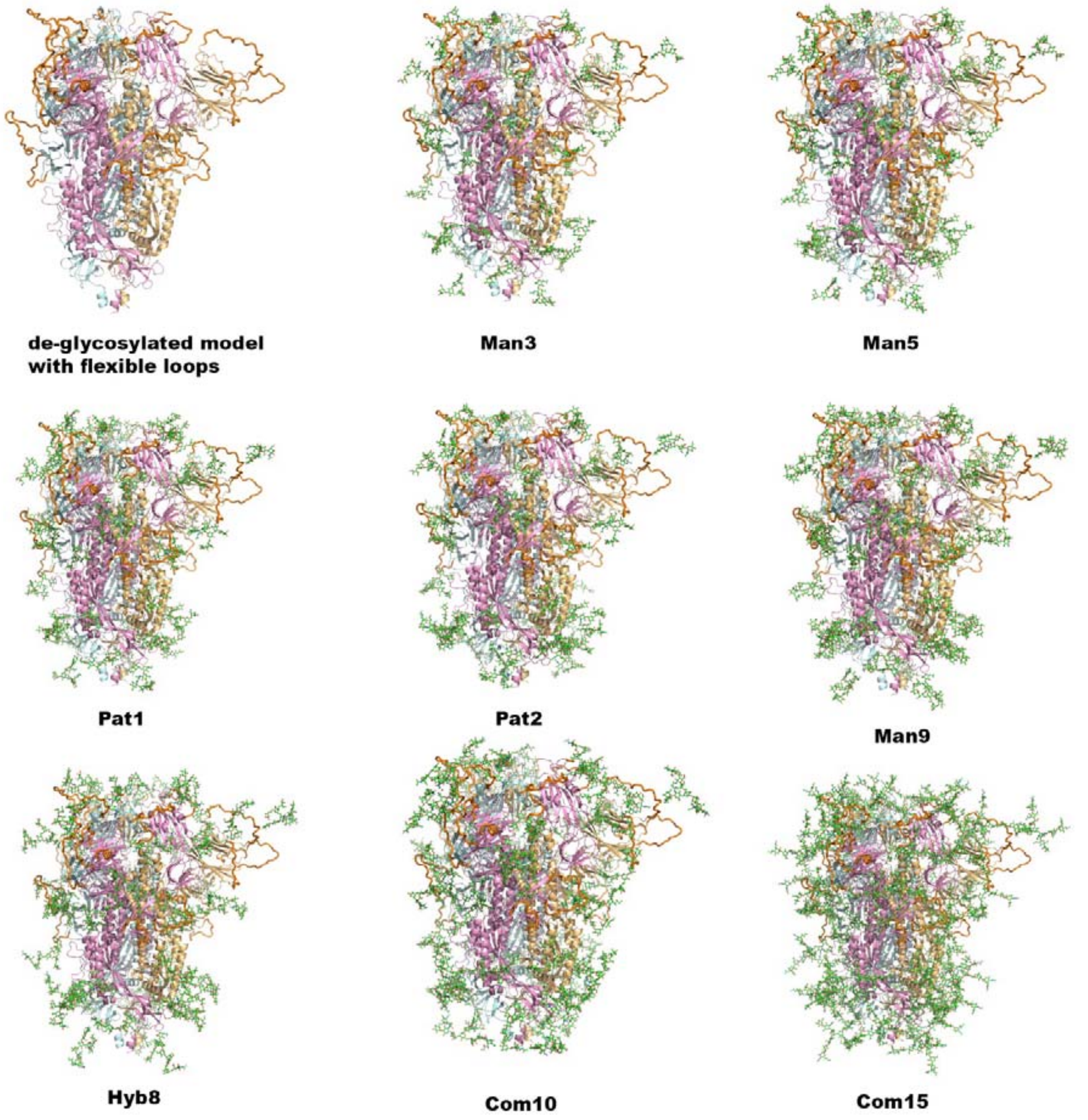

Supplementary Fig. 3. 3D illustration of the SARS-CoV-2 S trimeric spike with the addition of the flexible loops and various N-glycosylation patterns. 3D rendition of the SARS-CoV-2 S-protein trimeric spike without the flexible loops, with the flexible loops and with progressively larger homogenous and heterogeneous glycan chains.

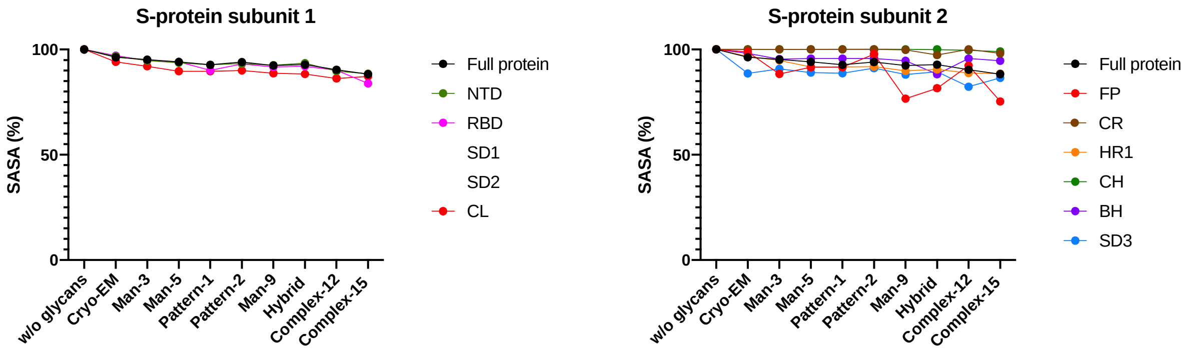

Supplementary Fig. 4. The solvent accessible surface area is not greatly affected by the addition of glycan chains. SASA for a solvent equal to the size of a H_2O molecule along the structural domains of the S-protein using increasingly larger homogeneous glycan chains, 2 heterogeneous glycosylation patters, as well as two previously published glycosylation profiles labelled complex-12 and complex-15. SASA of each structural domain are shown as a percentage in relation to the de-glycosylated model. The SASA of the total protein was additionally plotted.
